# Recent Advances in Fluorescence Imaging of Traumatic Brain Injury in Animal Models

**DOI:** 10.3389/fmolb.2021.660993

**Published:** 2021-05-26

**Authors:** Fei Lu, Jiating Cao, Qinglun Su, Qin Zhao, Huihai Wang, Weijiang Guan, Wenjuan Zhou

**Affiliations:** ^1^ Department of Rehabilitation Medicine, The First People’s Hospital of Lianyungang, The First Affiliated Hospital of Kangda College of Nanjing Medical University, Lianyungang, China; ^2^ Department of Chemistry, Capital Normal University, Beijing, China; ^3^ State Key Laboratory of Chemical Resource Engineering, College of Chemistry, Beijing University of Chemical Technology, Beijing, China

**Keywords:** traumatic brain injury, inflammation, molecular diagnostics, biomarkers, nanomaterial, imaging

## Abstract

Traumatic brain injury (TBI) is one of the top three specific neurological disorders, requiring reliable, rapid, and sensitive imaging of brain vessels, tissues, and cells for effective diagnosis and treatment. Although the use of medical imaging such as computed tomography (CT) and magnetic resonance imaging (MRI) for the TBI detection is well established, the exploration of novel TBI imaging techniques is of great interest. In this review, recent advances in fluorescence imaging for the diagnosis and evaluation of TBI are summarized and discussed in three sections: imaging of cerebral vessels, imaging of brain tissues and cells, and imaging of TBI-related biomarkers. Design strategies for probes and labels used in TBI fluorescence imaging are also described in detail to inspire broader applications. Moreover, the multimodal TBI imaging platforms combining MRI and fluorescence imaging are also briefly introduced. It is hoped that this review will promote more studies on TBI fluorescence imaging, and enable its use for clinical diagnosis as early as possible, helping TBI patients get better treatment and rehabilitation.

## Introduction

Traumatic brain injury (TBI) refers to a brain damage caused by trauma, usually occurring in traffic accidents, falls, violent blows, sports, and combat (Leeds et al., 2014; Treble-Barna et al., 2017; Li et al., 2018). As one of the top three specific neurological disorders worldwide, TBI has become a huge public problem that threatens human health and life. Currently, more than 50 million people suffer from TBI every year, which puts a heavy burden on their families and the whole society (Maas et al., 2017). During the TBI process, the initial impact causes both primary and secondary injuries. Primary injuries include cerebral concussion, cerebral contusion, laceration, and penetrating wounds that occur immediately as a result of direct mechanical damage (Katzenberger et al., 2013; Kwon et al., 2016; Barbacci et al., 2017). On the other hand, some pathophysiological processes, such as post-traumatic neurotransmitter release, free radical generation, mitochondrial dysfunction, inflammatory response, abnormal coagulation function, and blood−brain barrier damage, subsequently cause secondary brain injuries and lead to cerebrovascular and neurological disorders (Brown et al., 2019; Glotfelty et al., 2019; Ghosh et al., 2020). Therefore, rapid and sensitive imaging of brain tissues, cerebrovascular vessels, and cells is particularly important for the diagnosis and treatment of TBI.

Medical imaging including computed tomography (CT) and magnetic resonance imaging (MRI) is the most used imaging modality for TBI (Brody et al., 2015; Shin et al., 2018; Lindberg et al., 2019). CT is capable of objectively reflecting the size, shape, and distribution of brain tissues, while MRI can provide a higher level of anatomical detail of brain tissues for noninvasive and longitudinal assessment of vessel occlusion, tissue injury, and hemodynamics (Kim and Gean, 2011; Bouts et al., 2017). However, the radiation and carcinogenic risks to the CT examiners cannot be ignored, especially for special populations such as pediatric patients (Brix and Nekolla, 2012). Moreover, challenges remain in the MRI technology concerning the scanning protocols (e.g., spatial vs. temporal resolution), analytical approaches, contrast agents, and sensitivity (Lelyveld et al., 2010; Li et al., 2018). Therefore, the development and application of new imaging techniques for TBI is of great interest.

**SCHEME 1:**
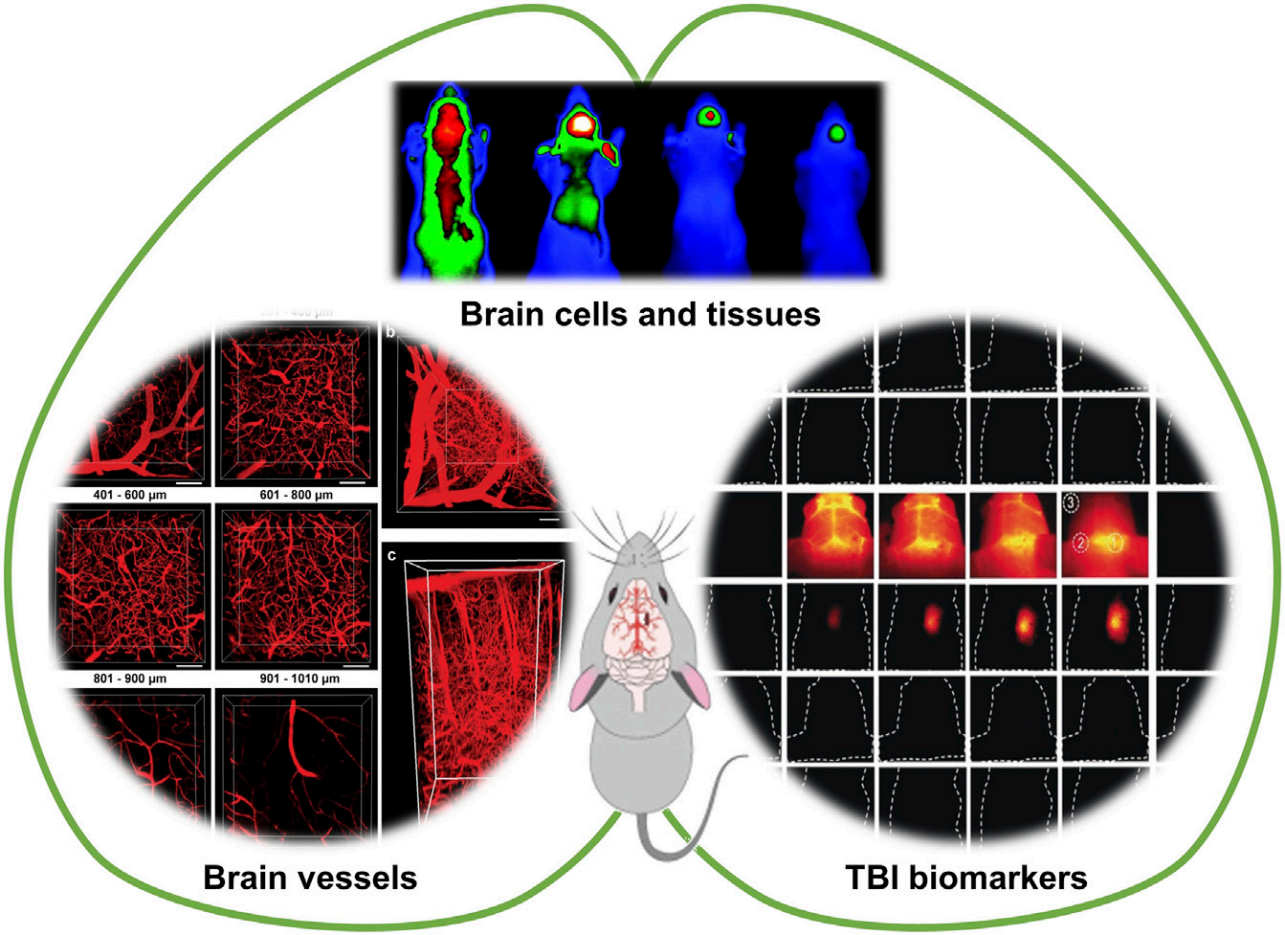
Fluorescence imaging for visualizing cerebral vessels, brain tissues and cells, and TBI-related biomarkers. Partially cited from ref (Li et al., 2018), ref (Wang et al., 2019), ref (Xie et al., 2013), ref (Maas et al., 2017).

Fluorescence imaging has attracted increasing attention in biological imaging because of its high spatial and temporal resolution, remarkable contrast, sensitivity, simplicity, and noninvasiveness (Ozawa et al., 2013; Liu et al., 2018; Li et al., 2020; Shah et al., 2020). With the rapid development of optical technology in the past two decades, the resolution of fluorescence imaging has experienced a dramatical improvement and reached up to the single nanometer scale (Wöll and Flors, 2017; Wilson et al., 2020; Zhang et al., 2020). The probes and labels employed for fluorescence imaging have also flourished, offering excitation ranges from single photon to two and even three photons, while the emission window has been extended to the near-infrared II (NIR-II, 1000–1700 nm) region (Wolfbeis, 2015; Li et al., 2019; Deng et al., 2020; Ji et al., 2020; Liu et al., 2020; Liu et al., 2021; Yang et al., 2021). Herein, we review recent advances in fluorescence imaging as a promising technique for the diagnosis and evaluation of TBI. To be specific, this review summarizes the current utilization and performance of fluorescence imaging for visualizing cerebral vessels, brain tissues and cells, and TBI-related biomarkers (Scheme 1). The design strategies for TBI imaging since 2008 are described and discussed in detail. Additionally, multimodal imaging platforms based on the combination of MRI and fluorescence imaging for the detection of TBI are also briefly presented. Our goal is to help researchers stay abreast of current advances of TBI fluorescence imaging and understand the potential opportunities and challenges.

## Imaging of Cerebral Vessels

In patients with craniocerebral injury, cerebral ischemia is the most common pathological change in secondary brain injuries, and is caused by the immediate decrease in cerebral blood flow (CBF). Peri-contusion ischemia is suggested to be induced by vasoconstriction, microvascular compression, and cerebral microvascular obstruction. To achieve sufficient spatial and temporal resolution, earlier studies raised the utility of *in vivo* fluorescence microscopy (IVM) for the investigation of vascular activities and vessel diameters in the microcirculation after TBI (Schwarzmaier et al., 2010; Obenaus et al., 2017). For example, visualization of the microvessels was performed by intravenously injecting fluorescein isothiocyanate-dextrane (FITC-dextrane) as the fluorescent plasma marker (Schwarzmaier et al., 2010). Meanwhile, white blood cells and platelets were stained with the fluorescent rhodamine 6G. With the help of fluorescent dyes with different emitting colors, multiple parameters of the microcirculation (e.g., vessel diameter, leukocyte-endothelial interactions, and microthrombus formation) can be analyzed in the same vessel segment.

The challenges of *in vivo* fluorescence imaging include light absorption and scattering, autofluorescence, and low depth penetration. To overcome these obstacles, near-infrared (NIR, 650–1700 nm) fluorescence imaging techniques, especially NIR-II (1000–1700 nm) fluorescence imaging, have been developed successively. The development of NIR fluorophores is closely related to the application of NIR fluorescence imaging in biological and medical fields. An effective strategy for constructing NIR fluorophores is to incorporate donor–acceptor–donor structures to reduce the band gap of fluorophores. For instance, a NIR-II fluorophore (IR-E1) was designed with benzo[1,2-c:4,5-c′]bis([1,2,5]thiadiazole) (BBTD) as the acceptor and thiophene-based moiety as the donor (Zhang et al., 2016). Under 808 nm excitation, IR-E1 showed NIR-II emission at 1071 nm, which was applied to *in vivo* cerebral imaging of hypoperfusion in a TBI mouse model. Compared to conventional fluorescence imaging, NIR-II fluorescence imaging allows dynamic *in vivo* imaging of the brain without craniotomy.

Besides the NIR emission, NIR excitation can also be used for deep tissue imaging. Two-photon fluorescence (2PF) imaging is usually performed by two-photon NIR excitation, which is a nonlinear process with a square dependence on the intensity of excitation light, allowing for three-dimensional (3D) tissue imaging with high spatial and temporal resolution. Meanwhile, the low-energy two-photon NIR excitation light has less damages to the tissues and deeper penetration depth. Schwarzmaier et al. (Schwarzmaier et al., 2015) applied *in vivo* two-photon microscopy to investigate vascular leakage in a clinically relevant model of TBI via green fluorescent protein (GFP) expression in vascular endothelial cells and intravenous injection of fluorescent plasma marker tetramethylrhodamine-dextran (TMRM). Arterioles and venules can be distinguished based on the levels of GFP expression. A penetration depth of 300 µm was achieved through the cranial window.

In addition, organic fluorophores with large multiphoton absorption cross section and high fluorescence quantum efficiency are capable of achieving both NIR excitation and emission. For example, Liu group developed an ultrasmall single-chain conjugated polymer dots (CPdots) with NIR-II excitation and bright NIR-I (700–950 nm) emission for deep *in vivo* two-photon fluorescence imaging of intact mouse brain (Wang et al., 2019). The vasculature was labeled by retro-orbital injection of CPdots, followed by 2PF imaging of brain blood vessels under 1200 nm fs laser excitation. With a cranial window, the maximal imaging depth reaches 1010 µm. Moreover, Tang group developed three-photon fluorescence (3PF) microscopy imaging technique for the *in vivo* brain vascular imaging by using a far-red/near-infrared (FR/NIR) luminogen (BTF) with remarkable aggregation-induced emission (AIE) characteristics (Qin et al., 2020). Through the further construction of BTF-based nanodots with a large three-photon absorption cross section, *In vivo* 3PF images and 3D high-resolution images of the mouse brain vessels with intact skull was obtained before/after brain thrombosis. Undoubtedly, these pioneering studies have a great potential for clinical applications.

## Imaging of Brain Cells and Tissues

TBI could induce the blood–brain barrier (BBB) disruption and neuroinflammations via regulating the lipid peroxidation and induction of oxidative stress to induce cell death and further disability of patient as the results of the secondary injury of TBI (Li et al., 2020). Observation or tracking of brain cells and tissues promote deeper understanding of injury mechanism, providing guidance for the prognosis and treatment of TBI. Various kinds of fluorescent labels including fluorescent proteins, small molecules, and nanoparticles have been developed for the fluorescence imaging of brain cells and tissues.

Neuroinflammatory responses (e.g., microglia/macrophage activation) could be induce by TBI, which is regarded as a key factor in the secondary injury cascade following TBI. Immunofluorescence staining is a classic method to investigate the mechanism of TBI-induced neuroinflammatory responses (Readnower et al., 2010; Villapol et al., 2017; Takahata et al., 2019; Mao et al., 2021). By double-labeling immunofluorescence, the levels of the lipid peroxidation marker 4-hydroxynonenal (4-HNE) and the protein nitration marker 3-nitrotyrosine (3-NT) in brain sections after exposure to blast have been determined (Readnower et al., 2010). The temporal course of brain oxidative stress following exposure to blast was obtained, which was rapidly increased at 3 h postexposure and were resolved by 24 h postexposure. The activation of microglial/macrophages could also be observed using double-labeling technique with two primary antibodies (polyclonal anti-rabbit P2Y12 for microglial cells and polyclonal anti-rat F4/80 for macrophages), and then corresponding fluorescent-dye conjugated secondary antibodies (anti-rabbit Alexa Fluor 568-conjugated IgG and anti-rat Alexa Fluor 488-conjugated IgG) (Villapol et al., 2017).

Another efficient methodology for the visual analysis of TBI is fluorescence protein expression. Yellow fluorescent protein (YFP) has been expressed under the promoter for the classically activated (M1) and alternatively activated (M2) macrophages for the identification of macrophage subset, demonstrating the heterogeneous polarization of the macrophage response to TBI (Hsieh et al., 2013). If YFP is expressed cortical neurons, the fluorescent protein can be used for the assessment of axonal injury over time within a well-defined axonal population, enabling an evaluation of the axonal injury pathobiology induced by TBI (Hånell et al., 2015). Moreover, fluorescent protein-expressing mesenchymal stem cells (MSCs) can be used for the location tracking of the MSCs during the TBI recovery progress (Hung et al., 2010; Lam et al., 2013).

Cerebral cell death is the major neuropathological basis in TBI, and apoptosis and autophagic cell death account for a considerable proportion. Molecular imaging for selective detection of apoptosis in experimental TBI was reported as early as 2008 (Reshef et al., 2008). Following intravenous administration *in vivo*, the animals with TBI were sacrificed, and brain tissues labelled with the apoptosis-sensitive N,N-didansyl-L-cystine (DDC) can be imaged via fluorescent microcopy. In addition, whole-body fluorescence imaging of cell death could be achieved using NIR fluorescent probes in a mouse model of TBI (Smith et al., 2012; Xie et al., 2013). A NIR fluorescent conjugate of a synthetic heat shock protein-90 (Hsp-90) alkylator, (4-N-S-glutathionylacetyl amino) phenylarsonous acid (GSAO), was utilized for labeling of apoptotic and necrotic cells (Xie et al., 2013). The GSAO can covalently bind with the Cys597 and Cys598 residues of Hsp-90 in mammalian cells through the cross-links of As(III) atom of GSAO and sulphur atoms of Hsp-90, Cys597, and Cys598. For healthy individuals, GSAO exists in the extracellular environment and is largely unreactive because there are few appropriately spaced cysteines thiols. When the plasma membrane is damaged (mid-to late-stage apoptotic cells), GSAO could enter the cell freely and display high reactivity. The selectivity of the fluorescent probe for dying and dead cells provides high signal-to-noise ratio and reliability for *in vivo* imaging of brain lesion cell death. Moreover, multiple biochemical changes in the early stage of TBI can be reported by using multiple probes in a single animal (Smith et al., 2012). A binary mixture of a NIR fluorescent probe (PSS-794) for detecting cell death and a deep-red dye (Tracer-653) for monitoring BBB disruption was described for multicolor imaging of cell death and blood-brain-barrier permeability in a single animal.

## Imaging of Biomarkers

Medical imaging techniques hardly provide an accurate prediction of the effects of brain injury (secondary injury) due to long-term impacts and heterogeneous nature of TBI (Mondello et al., 2011; Maas et al., 2017; Mondello et al., 2018). Biomarkers of brain injury refer to substances that can be detected and released into the cerebrospinal fluid and blood during brain injury. The level of biomarkers changes in the early stage of brain injury, which plays a crucial role in predicting early brain injury, identifying brain injury areas, and evaluating prognosis (Wang et al., 2018). Generally, brain injury is usually associated with neuroinflammation or nerve damage, which produces a number of associated biomarkers, such as acidity (pH) change, hypochlorous acid (HOCl), peroxynitrite (ONOO^−^), and calproteinase-1 (Zhai et al., 2019; Kudryashev et al., 2020; Li et al., 2020; Song et al., 2020). The sensitivity and specificity of biomarker detection are often more advantageous than imaging examination.

Neuroinflammation as one of the earliest hallmark features of TBI can cause an increased oxygen consumption and a hypoxic state in BV-2 cells. A dramatic decrease in mitochondrial pH appears as a result of cellular anaerobic respiration. To monitor pH changes, a ratiometric fluorescence probe (FRET-pH) was developed by covalently linking 6-hydroxy-quinoline-2-benzothiazole (ADN) as a fluorescent donor to a derivative of Rh6G (SRhB) as a fluorescent acceptor and a response group (Zhai et al., 2019). The fluorescence of ADN (λ_em_ = 454 nm) could be excited by absorption of one photon (λ_abs_ = 350 nm) or simultaneous absorption of two photons (λ_abs_ = 700 nm). SRhB exhibited intense orange-red fluorescence (λ_em_ = 562 nm) through energy transfer from AND and was highly sensitive in the pH range of 4.6–7.4. FRET-pH was able to clearly detect pH changes in both BV-2 cells and rat brain tissues using 2PF microscopy.

TBI-associated neuroinflammation can also cause sustained oxidative stress (OT) to produce reactive oxygen species (ROS), including HOCl, ONOO^−^, etc. The general strategy for detecting mitochondrial ROS is similar to that for detecting mitochondrial pH. For example, Liu et al. synthesized a ratiometric two-photon fluorescence probe (Mito-P-OCl) consisting of three moieties: a rhodanol moiety (Rhod-c), a dihydrazide moiety, and a quaternized pyridine moiety (Song et al., 2020). They acted as the two-photon fluorophore, the HOCl response group, and the mitochondrial-targeting group, respectively. The as-prepared Mito-P-OCl itself had blue fluorescence due to the occurrence of excited-state intramolecular proton transfer (ESIPT) in the molecule. In the presence of HOCl, the rhodol ring on Mito-P-OCl could be opened to form Rhod-c, in which the ESIPT process was inhibited, thus showing a strong red fluorescence. Taking advantages of the rhodol ring-opening/ring-closing switch, Mito-P-OCl successfully achieved the monitoring of endogenous HOCl in living cells and brain tissue. To further expand the above strategy to *in vivo* imaging, a novel targeted activatable NIR-II nanoprobe (V&A@Ag_2_S) with emission at the range of 1000–1800 nm was designed and synthesized (Li et al., 2020). The V&A@Ag_2_S includes three components: VCAM1 binding peptide (VHPKQHR) for targeting the inflamed endothelium expressing VCAM1 in TBI regions, a NIR absorber A1094 for responding ONOO^−^ changes, and Ag_2_S QD for emitting NIR-II fluorescence. Due to the large overlap between the absorption spectrum of A1094 and the emission spectrum of Ag_2_S QD, the fluorescence of V&A@Ag_2_S is quenched through the energy transfer from Ag_2_S QD to A1094. On the contrary, the presence of ONOO^−^ oxidized A1094 to decrease the absorbance at 1094 nm, turning on fluorescence signal of the Ag_2_S QD at 1050 nm. The unique optical properties of NIR-II imaging enabled real-time dynamic measurement of ONOO^−^ in live mice with brain vascular injury.

## Multimodal Imaging

**FIGURE 1:**
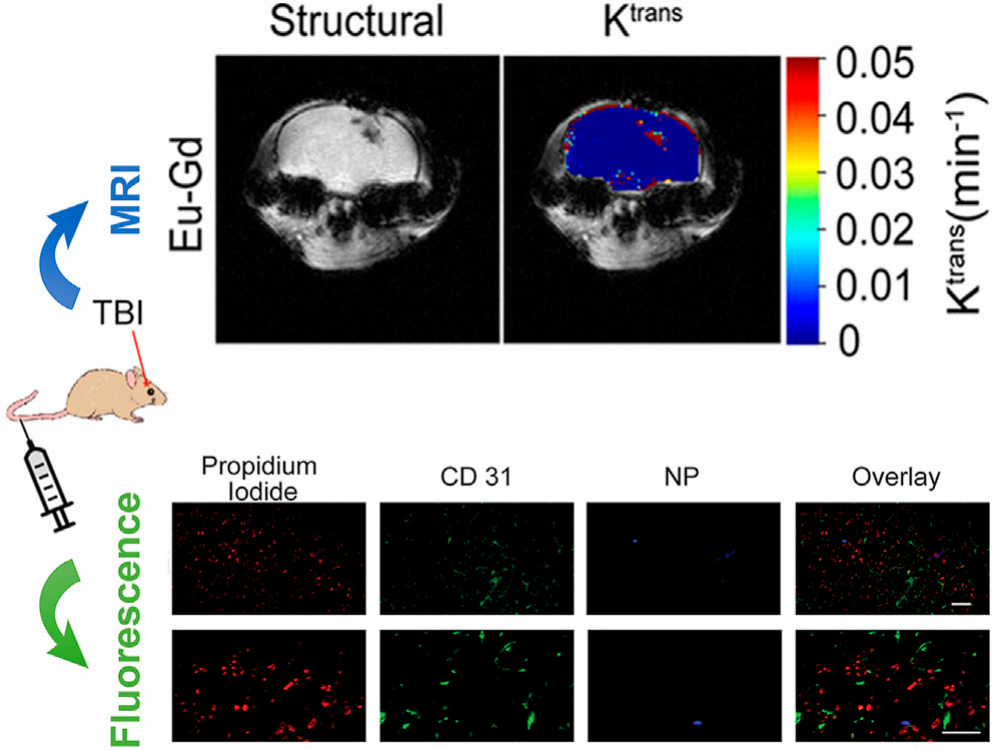
Multimodal fluorescence and magnetic resonance imaging of passive accumulation and retention in a mouse traumatic brain injury model. Adapted and modified with permission from ref (Bony et al., 2020) (Copyright 2020 American Chemical Society).

Medical imaging including CT, MRI, X-ray is the most used imaging modality for TBI without any surgery (James and Dasarathy, 2014; Du et al., 2016; Kaur and Singh, 2020). Due to different imaging principles, a single imaging modality often has limitations in terms of sensitivity, specificity, targeting ability, and spatial resolution. Multimodal imaging probes can provide diagnostic information combining different imaging modalities, which overcomes the deficiency of traditional single-modal imaging, and widens the application range of imaging technology. Multimodal imaging enables rapid and accurate imaging at specific target sites to provide a comprehensive assessment of functional, structural, and metabolic changes *in vivo* (Feng et al., 2019). Therefore, the development of multimodal probes for TBI has become the focus of research (Guo et al., 2019; Bony et al., 2020; Schomann et al., 2020). Among them, fluorescence/MRI bimodal probes have attracted much attention with the superior advantages of high tissue resolution and imaging sensitivity. A feasible attempt is to use the mixed lanthanide oxide magnetic nanoparticles (MNPs) containing europium (Eu) for fluorescence imaging and gadolinium (Gd) for MRI in TBI (Bony et al., 2020). Moreover, these Eu–Gd NPs can be modified with different functional poly(ethylene glycol) (PEG) to not only tune their hydrodynamic dimensions and surface charge, but also to improve targeting ability and biocompatibility. In a controlled cortical impact (CCI) mouse model of TBI, MRI data showed that Eu-Gd NPs were rapidly accumulated and retained in the mouse brain after intravenous injection, while fluorescence imaging revealed their spatial distribution on cells and tissues (Figure 1). It is worthwhile to expect that more NIR and multiphoton fluorophores suitable for tri-modal or even quad-modal imaging can be designed and synthesized TBI.

## Conclusion and Outlook

Over the past decade, various fluorescence imaging techniques for TBI diagnosis have made considerable progress due to their abilities to directly detect and visualize brain microstructures (e.g., blood vessels, tissues, and cells) and to track dynamic changes during TBI injury, treatment, and rehabilitation. It overcomes the deficiency of strong radiation, low resolution and low sensitivity of conventional brain MRI and CT, showing great clinic potentials in the diagnosis and treatment of TBI. Superior to conventional fluorescence imaging in the visible and NIR-I spectral range (400–900 nm), NIR II fluorescence imaging greatly reduces tissue scattering, light absorption, and autofluorescence, allowing deeper tissue penetration, higher spatial resolution, and dynamic *in vivo* imaging of the brain without craniotomy. In addition, the appearance of organic fluorophores with large photon absorption cross sections and high fluorescence quantum efficiency has also greatly promoted the development of two-photon or even three-photon imaging for TBI diagnosis. With the continuous development of fluorescence imaging technology, researchers have begun to explore novel multimodal probes (e.g., fluorescence/MRI dual-modal probe) to achieve complementary parameters, so as to make more accurate diagnosis and effective treatment of TBI.

Notably, challenges remain in translating the TBI fluorescence imaging platform from the research setting to more practical devices and clinical applications. Hence, more investigations and innovations are necessary to develop universal fluorescent dyes, improve the operability of the method, and reduce professional and technical requirements. NIR II or multi-photon fluorescence imaging can be regarded an ideal candidate for *in vivo* and *in situ* imaging of brain. In order to achieve full-scale and high-quality imaging of the brain through the scalp and skull, fluorophores with higher quantum yield should be designed and developed. Another promising strategy is the combining of fluorescence imaging with other imaging techniques (e.g., MRI, CT, and X-ray). The multimodal imaging system can provide a more accurate and comprehensive reference for the diagnosis and treatment of TBI, especially for the secondary brain injury after TBI. In addition, the neurotoxicity of fluorescent probes must be considered when performing brain imaging. The effects of the developed fluorescent probes on human health and brain function are unclear, which also limits the pace of clinical applications of fluorescent imaging.

Compared with brain structure (blood vessel, tissue, etc.) imaging, the identification and detection of TBI-associated biomarkers can provide a more accurate molecular level diagnosis of TBI, which is the key to the early diagnosis of craniocerebral injury. The identified biomarkers allow us to measure the extent of damage and monitor the recovery process from brain injury. It is worth noting that the biomarkers released at different time periods of the occurrence and development of TBI are different, thus further explore about the optimum detection moment for different types of biomarkers is of great significance in assessing the injury and prognosis of patients with TBI.
